# Epidemiological, clinical and therapeutic profile of hepatocellular carcinoma in Toamasina Madagascar

**DOI:** 10.3332/ecancer.2026.2130

**Published:** 2026-05-26

**Authors:** Randriamanovontsoa Niaina Ezra, Ranaivomanana Mampionona, Rasataharifetra Hanta, Jano, Rabenjanahary Tovo Harimanana, Rafaramino Florine

**Affiliations:** 1 Faculty of Medicine, University of Toamasina, Toamasina 501, Madagascar; 2 Faculty of Medicine, University of Fianarantsoa, Fianarantsoa 301, Madagascar; 3 Faculty of Medicine, University of Antananarivo, Antananarivo 101, Madagascar

**Keywords:** alpha-fetoprotein, hepatocellular carcinoma, viral hepatitis, Madagascar

## Abstract

**Background:**

International recommendations for the management of hepatocellular carcinoma (HCC) are often difficult to apply in low-resource settings. This study aimed to describe the epidemiological, clinical, diagnostic and therapeutic characteristics of HCC patients managed at Analankininina University Hospital in Toamasina, Madagascar.

**Methods:**

This retrospective, descriptive cross-sectional study was conducted from January 1, 2017 to December 31, 2021. Diagnosis of HCC was based on histological confirmation or non-invasive radiological criteria (arterial enhancement with washout on dynamic imaging) and/or elevated alpha-fetoprotein >500 ng/ml in cirrhotic patients. Serological, radiological and therapeutic data were analysed. The Child–Pugh score was used to assess liver function and the Barcelona Clinic Liver Cancer (BCLC) classification was used for staging and therapeutic guidance. Alcoholism was defined as documented chronic excessive alcohol consumption recorded in medical files.

**Results:**

Forty-three patients were included. Mean age was 52.57 ± 19.74 years. The male-to-female ratio was 2.31. Most patients were diagnosed at advanced stages (BCLC C/D: 39/43). Alpha-fetoprotein was >500 ng/ml in 33/43 patients. Liver ultrasound was performed in all cases, CT scan in 9/43 and histological confirmation was obtained in 9/43. Symptomatic palliative care was administered in 33/43 patients.

**Conclusion:**

HCC is frequently diagnosed at an advanced stage in our setting, limiting curative treatment options. Limited access to dynamic imaging, histopathology and systemic therapies contributes to suboptimal management. Strengthening hepatitis B virus birth dose vaccination, early screening of at-risk populations and improving access to diagnostic tools are essential priorities.

## Introduction

Hepatocellular carcinoma (HCC) is the most frequent form of primary liver cancer and a major public health problem worldwide. It most often occurs in a diseased liver, particularly a cirrhotic one, linked to chronic liver diseases such as infections by hepatitis B (HBV) and C (HCV) viruses, chronic alcoholism or metabolic steatosis.

While industrialised countries benefit from high-performance diagnostic tools and multidisciplinary therapeutic strategies, the situation is much more worrying in developing countries, where the burden of the disease is particularly heavy. In this context, the difficulties are multiple: limited access to high-resolution imaging, insufficient systematic screening programs, frequent diagnostic delays and low resources for treatment. Moreover, low awareness among at-risk populations, a lack of specialised infrastructure and the high cost of innovative treatments worsen the inequalities in care.

In Madagascar, infection with HBV and HCV viruses remains endemic. Chronic alcoholism and metabolic liver disorders also contribute to the increasing incidence of this cancer. Early diagnosis is rare due to the absence of national targeted screening programs and limited access to specialised biological and radiological examinations. Consequently, the majority of patients are diagnosed at an advanced stage. Specialised surgical interventions are rarely available, liver transplantation does not exist and locoregional treatments are not very accessible. Expensive and non-reimbursed systemic treatments make their use marginal.

This work aims to describe the epidemiological, clinical and therapeutic characteristics of patients with HCC in a university hospital in Toamasina, Madagascar, with a view to proposing avenues for improvement adapted to local realities.

## Method

This was a retrospective, descriptive and cross-sectional study carried out on medical records, from 1st January 2017, to 31st December 2021, in the Internal Medicine, Intensive Care Unit, Visceral Surgery and Oncology departments of the Analankininina University Hospital (Centre Hospitalier Universitaire (CHU)) in Toamasina, Madagascar. The records of patients who met the diagnosis of HCC according to one of the following inclusion criteria were selected:

- Histological confirmation of HCC from a liver biopsy.
  OR
- On ultrasound, CT scan or abdominal magnetic resonance imaging (MRI), the presence of one or more hypervascularised nodules greater than 1 cm in the arterial phase is associated with ‘washout’ in the portal and/or late phase.
  AND/OR
- Cirrhotic patient with a single or multiple nodular liver associated with an elevation of Alpha-fetoprotein (AFP) >500 ng/ml.

The positive diagnosis of cirrhosis was based on clinical and abdominal ultrasound findings. The suggestive clinical signs were hepatomegaly with a firm, hard, sharp lower border liver, splenomegaly, ascites, digestive hemorrhage related to the rupture of esophageal varices, asterixis and behavioural disorders. Ultrasound signs included dysmorphic liver, heterogeneous echotexture with regeneration nodules, splenomegaly, dilatation of the portal vein and the presence of collateral varices and ascites.

The following factors were studied.

- Social and behavioural risk factors:
  - Gender,
  - Age,
  - Occupation sector: primary (agriculture and fishing), secondary (industrial activities), tertiary (services such as trade and administration) and others (unemployed, students and unspecified).
  - Consumption alcoholism was defined as documented chronic excessive alcohol consumption recorded in the medical files and assessed by the treating physician. Patients were classified as alcoholic when chronic consumption exceeded 30g/day for men and 20g/day for women, according to international hepatology guidelines.
- Clinical factors: circumstances of discovery, performance status index (PSI)
- Serological and anatomopathological factors: serology for HBV and HCV infection, AFP, anatomopathological result and Child–Pugh score. Child-Pugh score was used to assess liver function severity and prognosis (class A, B or C)
- Radiological factors: liver ultrasound, CT scan or MRI.
- Therapeutic factors: Barcelona classification and the type of treatments received. The Barcelona Clinic Liver Cancer (BCLC) classification was used to stage the disease and guide therapeutic decision-making.

Descriptive statistical analysis was performed using Microsoft Excel^®^ 2016. Continuous variables were expressed as mean ± standard deviation or median as appropriate. Categorical variables were expressed as proportions (n/N). This retrospective study was conducted in accordance with institutional ethical standards and the Declaration of Helsinki. Patient confidentiality and anonymity were strictly maintained.

## Results

In total, 43 patient files were selected for this study. The meanage was 52.57 ± 19.74 years. Age distribution showed no major skewness. The majority of patients were aged between 50 and 69 years (24/43). The male-to-female ratio (M:F) was 2.31(30/13). Regarding occupation, 14/43 patients were involved in the primary sector (agriculture and fishing), 9/43 in the secondary, 11/43 in tertiary sector and 9/43 were classified as others (Table 1).

The most frequent circumstances of discovery were: right hypochondrial pain (36/43), ascites (24/43) and impaired general condition (22/43). The signs could be isolated or associated. Alcoholism, defined as documented chronic excessive alcohol consumption, was reported in 29/43 patients. Most patients presented with an altered general condition, with 25/43 classified as performance status III or IV.

On a biological level, serologies for HBV and HCV or a HBV/HCV co-infection were respectively: 14/43, 5/43 and 4/43. AFP was elevated more than 500 ng/ml in 33/43. According to the Child–Pugh classification, 24/43 were class B and 10/43 class C.

On an iconographic level, all patients had benefited from a liver ultrasound; a CT scan was performed in 9/43 patients and no patient was able to undergo an MRI. Cirrhotic features were observed in 22/43 cases. Tumour size was greater than 20 mm in 41/43. Multiple nodules were present in 25/43.

Histological confirmation was obtained in 9/43 cases.

According to the BCLC classification, 33/43 patients were classified as stage D. Curative treatement (partial hepatectomy) was performed in 3/43 patients. Thyrosine kinase inhibitors were administred in 7/43; symptomatic palliative care was provided to 33/43 cases.

Table 2 shows the details of these clinico-biological, radiological and therapeutic characteristics.

## Discussion

### Epidemiological profile

This study highlights several epidemiological characteristics of the HCC cases observed over a given period. The population studied is predominantly male (M:F = 2.31) (69.77%), which is consistent with trends observed at the national, African and global levels. Ranaivomanana *et al* [1], in a study at CHU Tambohobe Fianarantsoa, reported six cases, four of which were men. This male predominance was also observed (69.05%) in a study conducted by Rakotozafindrabe *et al* [2] at the Joseph Raseta Befelatanana Hospital, Antananarivo. According to data from the World Health Organisation (WHO) and the International Agency for Research on Cancer, HCC affects men two to four times more often than women [3, 4]. This male predominance could be explained by greater exposure to risk factors such as chronic alcoholism, smoking, consumption of food contaminated with aflatoxins and chronic viral hepatitis, especially hepatitis B, which is widespread in sub-Saharan Africa [5]. Hormonal and behavioural factors could also play a role in this disparity [6].

Patients aged 50 to 69 constitute the majority of cases (24/43; 55.81%) with an average age of 52.57 ± 19.74 years, which corresponds to the average age of onset of HCC in many developing countries. In Africa, the disease generally appears at a younger age than in Europe or North America, due to early infection with the HBV, often transmitted perinatally or during early childhood [7, 8]. In Madagascar, epidemiological data remain limited, but the trend of onset at an intermediate or advanced age is shown in the studies by Ranaivomanana *et al* [1]at CHU Tambohobe Fianarantsoa (49 years) and that of Rakotozafindrabe *et al* [2] at the Joseph Raseta Befelatanana Hospital, Antananarivo (56.6 years).

The predominance of the primary sector (14/43) among those affected is significant. This sector includes farmers and fishermen, who are often exposed to aflatoxins, toxins produced by molds that grow on poorly stored food (peanuts, corn and rice). These substances are recognised as major risk factors for HCC, especially in association with chronic HBV infection [9, 10]. Furthermore, rural populations have limited access to healthcare, particularly hepatitis screening and vaccination, which contributes to the late diagnosis of HCC [11].

## Diagnostic challenges

The late diagnosis of HCC is a recurrent situation in low-resource countries. In the present study, nearly 91% of patients are diagnosed at an advanced stage (Barcelona stages C and D), with revealing clinical signs such as pain, ascites and impaired general condition. AFP greater than 500 ng/ml represented 33/43. This observation is consistent with the results of a study conducted in Antananarivo, where 98% of patients were diagnosed at an advanced stage [2]. Diagnostic delay is also well documented in sub-Saharan Africa: according to the Africa Liver Cancer Consortium, more than 90% of patients are diagnosed at a non-curable stage [12].

The recommended imaging to establish a non-invasive diagnosis was rarely performed. Liver ultrasound was performed in 100% of patients, while an abdominal CT scan was only performed in 20.93% of cases, and no MRI was performed. In addition, anatomopathological confirmation was only obtained in 20.93% (9/43 cases). The present study corroborates the results of the Ranaivomanana study in Tambohobe Fianarantsoa, where the CT scan was performed in 50%, while the biopsy was performed in 16.66% of cases [1]. These results highlight the diagnostic constraints and show a dependence on ultrasound in the absence of generalised access to second-line imaging techniques and histological confirmation.

Abdominal ultrasound is a common HCC screening tool, particularly in at-risk populations, due to its availability, relatively low cost and safety. It allows the detection of liver nodules as part of the surveillance of cirrhotic patients [13]. However, its diagnostic performance is highly operator-dependent and decreases in the face of small lesions or in the presence of hepatic steatosis [14]. Therefore, ultrasound alone does not allow for a certain confirmation of an HCC diagnosis, especially in the absence of typical signs. In addition, the ultrasound in our study was without an injected contrast medium. However, ultrasound without contrast enhancement has limited sensitivity for small lesions and early-stage tumours. Consequently, some cases may have been missed during the early disease stage or diagnosed only when lesions became clinically apparent. This diagnostic limitation may partly explain the high proportion of patients presenting at an advanced stage in our cohort. The lack of access to contrast-enhanced imaging techniques, such as triphasic CT scan or MRI, likely contributed to delayed diagnosis and reduced eligibility for curative treatments. This finding highlights the impact of resource-limited settings on HCC detection and emphasises the need for improved surveillance strategies among high-risk populations, particularly patients with chronic viral hepatitis.

International recommendations, particularly those of the European Association for the Study of the Liver and American Association for the Study of Liver Diseases, advocate the use of dynamic imaging such as CT or MRI with contrast medium injection to establish a non-invasive diagnosis of HCC. The key radiological criterion is based on the combination of arterial enhancement followed by ‘washout’ in the portal or late phase, which has high specificity for HCC [15, 16]. While the majority of cases had a tumour size greater than 2 cm, the absence of dynamic imaging constitutes a significant limitation, likely to compromise early diagnosis, particularly in cases where the nodules do not have typical ultrasound characteristics.

Furthermore, the low rate of anatomopathological confirmation (9/43; 20.93%) reflects limited access to liver biopsy. In a context where dynamic imaging is insufficient or unavailable, biopsy remains essential to confirm the diagnosis [15, 17]. However, it is often avoided due to the risks of complications such as bleeding, tumour dissemination, cost and the lack of qualified human resources in disadvantaged environments [18]. In Toamasina and particularly in the Eastregion, MRI does not exist. Two CT scanners are present, one of which is housed in a public institution. The cost of this examination remains a constraint for most patients. Ultrasound is available to everyone, but very often without an injection. Liver biopsy is rarely performed in this region due to a lack of an adequate technical platform.

### Therapeutic situation

The diagnostic delay compromises curative options, limiting access to potentially effective treatments. A small proportion of patients had benefited from partial hepatectomy, 3/43 (6.98%). In the literature, surgery is a preferred option for patients with preserved liver function and a single tumour without vascular invasion or metastasis [15]. However, in our study population, cases were very often seen with nodules larger than 20 mm and multiple. The detection of early cases is very limited in our context. Rakotozafindrabe *et al* [2] reported a result comparable to our study; curative treatment represented only 2.38%.

The use of targeted therapies, particularly tyrosine kinase inhibitors like sorafenib, was observed in 7/43 cases. This modest rate can be attributed to the high cost of these treatments and their limited accessibility in the country. Sorafenib, the first systemic treatment approved for advanced HCC, modestly improves survival but remains expensive and its side effects are often poorly tolerated, which could limit prolonged use [19].

Finally, a significant proportion of patients 33/43 were managed only with symptomatic palliative care. This result is consistent with that observed at the Gastroenterology-Hepatology department in Befelatanana Antananarivo, where 97.62% of patients had received symptomatic palliative care [2]. This therapeutic choice is necessary due to the advanced stage of the disease, the patient’s altered general condition or the inaccessibility of curative and systemic treatments. In many African and Asian contexts, this orientation is frequent and highlights the need to strengthen prevention, screening and equitable access to treatment strategies [13, 20]. In total, conservative surgery can be performed within the hospital. Liver transplantation and all types of radiotherapy do not exist in the Region or throughout the country. Other loco-regional treatments such as radiofrequency or percutaneous ethanol injection, and chemoembolisation are not available. The use of targeted therapy and/or immunotherapy remains very limited due to the cost of these treatments. Very often, patients benefit from symptomatic palliative care.

### Suggestions

These results highlight the major diagnostic and therapeutic challenges in the management of HCC in a low-resource setting: diagnostic delay, insufficient infrastructure and the high cost of innovative treatments. This situation reiterates the importance of strengthening primary and secondary prevention actions.

Vaccination against the HBV is the main lever for primary prevention of HCC. The WHO recommends administering a dose at birth followed by two to three additional doses in the following months [21]. In Madagascar, HBV vaccination has been integrated into the Expanded Program on Immunisation since 2002, with coverage estimated at around 70% for the three doses, but the birth dose is not yet systematically administered [22, 23]. However, this dose is essential to prevent vertical transmission, the main source of chronic infection in countries with intermediate to high endemicity [24].

In addition to HBV, the other major factors of HCC are HCV infection, chronic alcoholism, aflatoxicosis, non-alcoholic steatohepatitis and metabolic diseases [25]. In Madagascar, as in several African countries, co-exposure to HBV and aflatoxin B1 is becoming particularly worrying [26]. It is therefore necessary to strengthen the universal administration of the birth dose of the HBV vaccine, secondary prevention by active screening of chronic HBV/HCV carriers and their antiviral treatment and the fight against alcoholism. The fight against aflatoxin involves improving agricultural and storage practices, an area still not well controlled in Madagascar despite the efforts of some agricultural projects [27].

The GALAD score allows for early diagnosis. It is composed of five parameters: gender, age, AFP, its L3 fraction and Des-gamma-carboxyprothrombin (DCP or PIVKA-II) and has been validated in several multicenter studies, showing a performance superior to that of AFP alone, particularly for the detection of early forms of HCC [28]. In a multicenter study conducted by Best *et al* [28] grouping patients from the United Kingdom, Germany and Japan, the GALAD score showed an area under the curve (AUC) of 0.97 for the diagnosis of HCC, compared to 0.76 for AFP alone. Another study by Singal *et al* [29], conducted in the United States, reported an AUC of 0.95 for GALAD in a cohort of cirrhotic patients, confirming its diagnostic superiority. A recent study by Nartey *et al* [30] in Ghana reported that the GALAD score has a sensitivity of 81% and a specificity of 86%. In Nigeria, Akinyemiju *et al* [31] evaluated the applicability of GALAD and reported good sensitivity, but logistics remain a challenge for its implementation. The GALAD score represents a promising tool in the early detection of HCC, with excellent diagnostic accuracy. Its implementation in health systems, however, requires a cost-effectiveness evaluation and adaptation to regional realities.

## Conclusion

This study provides insight into the epidemiological and clinical characteristics of HCC in a teaching hospital in Toamasina Madagascar, where patients predominantly present at advanced disease stages. Late diagnosis, limited diagnostic resources and restricted access to specialised treatments significantly influence therapeutic options and prognosis. Improving early detection represents the most effective strategy to reduce HCC mortality in this setting. Strengthening hepatitis prevention programs, implementing systemic surveillance for high-risk population and improving access to diagnostic imaging should be prioritised. Future health policies should focus on early screening, decentralisation of diagnostic facilities and capacity building in liver cancer management to improve patient outcomes in Madagascar.

## Conflicts of interest

No conflicts of interest to declare.

## Funding

This research was not supported by any specific grants from public, commercial or nonprofit funding agencies.

## Figures and Tables

**Table 1.** Sociodemographic characteristics of HCC patients at CHU Analankininina Toamasina.

Variables	n/43
Gender Male Female	30 13
Age (years) 11 months–29 30–49 50–69 70–89	5 8 24 6
Occupation sector Primary (agriculture and fishing) Secondary (industry) Tertiary (service) Others (unemployed/students)	14 9 11 9

**Table 2.** Clinico-biological, radiological and therapeutic characteristics of HCC patients at CHU Analankininina Toamasina.

Variables	n/43
Circumstances of discovery Right hypochondrial pain Ascites Impaired general condition Jaundice Weight loss Hepatomegaly Splenomegaly Others*	36 24 22 10 8 7 5 6
PSI 0/I/II III/IV	2/5/11 18/7
HBVand HCV serology HBV HVC HBV & HCV coinfection Indeterminate	14 5 4 20
AFP (ng/ml) <200 200–500 >500	4 6 33
Child-Pugh score A/B/C	6/27/10
Tumour size (mm) 1–3/30–50/> 50	8/12/23
Nodules:single/multiple	18/25
Barcelone classification 0 A/B C/D	0 3/1 6/33
Treatements Partial hepatectomy Tyrosine kinase inhibitor Symptomatic palliative care	3 7 33
(*): collateral venous circulation, digestive hemorrhage, hepatic encephalopathy.	

## References

[1] Ranaivomanana M, Rakotovao A, Fanantenantsoa R (2017). Étude préliminaire épidémio-clinique des carcinomes hépatocellulaires observés au Centre Hospitalier Universitaire Tambohobe Fianarantsoa. Revue Médicale De Madagascar.

[2] Rakotozafindrabe ALR, Razafindrazoto CI, Laingonirina DHH (2022). Demographic, clinical and aetiological characteristics of patients with hepatocellular carcinoma followed between 2012 and 2017 at University Hospital Joseph Raseta Befelatanana, Antananarivo, Madagascar. ecancer.

[3] World Health Organization (2022). Global Cancer Observatory: Liver cancer fact sheet.

[4] International Agency for Research on Cancer (IARC) (2022). Cancer Today: Liver Cancer Incidence and Mortality.

[5] Schweitzer A, Horn J, Mikolajczyk RT (2015). Estimations of worldwide prevalence of chronic hepatitis B virus infection: a systematic review. Lancet.

[6] Naugler WE, Sakurai T, Kim S (2007). Gender disparity in liver cancer due to sex differences in MyD88-dependent IL-6 production. Science.

[7] Yang JD, Hainaut P, Gores GJ (2019). A global view of hepatocellular carcinoma: trends, risk, prevention and management. Nature Rev Gastroenterol & Hepatol.

[8] Ott JJ, Stevens GA, Groeger J (2012). Global epidemiology of hepatitis B virus infection: new estimates of age-specific HBsAg seroprevalence. Vaccine.

[9] Liu Y, Wu F (2010). Global burden of aflatoxin-induced hepatocellular carcinoma: a risk assessment. Environ Health Perspect.

[10] Wild CP, Gong YY (2010). Mycotoxins and human disease: a largely ignored global health issue. Carcinogenesis.

[11] World Health Organization, Regional Office for Africa (2021). Hepatitis situation and response in the WHO African Region: Progress report 2021.

[12] Yang JD, Mohamed EA, Aziz AOA (2017). Characteristics and outcomes of hepatocellular carcinoma in Africa: the Africa Liver Cancer Consortium. Lancet Gastroenterol & Hepatol.

[13] Forner A, Reig M, Bruix J (2018). Hepatocellular carcinoma. Lancet.

[14] Singal AG, Mittal S, Yerokun OA (2017). Hepatocellular carcinoma screening in patients with cirrhosis in a US cohort. Cancer Epidemiology Biomarkers & Prevention.

[15] Galle PR, Forner A, Llovet JM (2018). EASL Clinical Practice Guidelines: management of hepatocellular carcinoma. J Hepatol.

[16] Singal AG, Llovet JM, Yarchoan M (2023). AASLD practice guidance on prevention, diagnosis, and treatment of hepatocellular carcinoma. Hepatology.

[17] Omata M, Cheng AL, Kokudo N (2017). Asia-Pacific clinical practice guidelines on the management of hepatocellular carcinoma: a 2017 update. Hepatol Int.

[18] Heimbach JK, Kulik LM, Finn RS (2018). AASLD guidelines for the treatment of hepatocellular carcinoma. Hepatology.

[19] Cheng AL, Kang YK, Chen Z (2009). Efficacy and safety of sorafenib in patients in the Asia-Pacific region with advanced hepatocellular carcinoma: a phase III randomised, double-blind, placebo-controlled trial. Lancet Oncol.

[20] Ginsburg O, Bray F, Coleman MP (2017). The global burden of women’s cancers: a grand challenge in global health. Lancet.

[21] Organization WH (2017). Hepatitis B vaccines: wHO position paper, July. Weekly Epidemiological Rec.

[22] Ministère de la Santé Publique à Madagascar (2022). Rapport annuel du Programme Élargi de Vaccination (PEV).

[23] UNICEF (2023). https://data.unicef.org/resources/immunization-dashboard.

[24] Hyams KC (1995). Risks of chronicity following acute hepatitis B virus infection: a review. Clin Infect Dis.

[25] Mcglynn KA, London WT (2011). The global epidemiology of hepatocellular carcinoma: present and future. Clinics Liver Dis.

[26] Andriamandimby SF, Rakoto-Andrianarivelo M, Randrianarisoa E (2021). Prevalence of hepatitis B and aflatoxin exposure among children in Madagascar. BMC Public Health.

[27] Kouadio JH, Dano DS, Meka S (2020). Strategies for reducing exposure to aflatoxin in Sub-Saharan Africa: a review. Afr J Food Sci.

[28] Best J, Bechmann LP, Sowa JP (2016). GALAD score detects early hepatocellular carcinoma in an international cohort of patients with nonalcoholic steatohepatitis. Gastroenterology.

[29] Singal AG, Tayob N, Mehta A (2017). Validation of the GALAD model for hepatocellular carcinoma detection in patients with cirrhosis. Gastroenterology.

[30] Nartey YA, Yang JD, Zemla TJ (2024). GALAD score for the diagnosis of hepatocellular carcinoma in Sub-Saharan Africa: a validation study in Ghanaian patients. Cancer Res Commun.

[31] Akinyemiju T, McDonald JA, Lazenby GB (2020). Disparities in hepatocellular carcinoma occurrence and outcomes among African Americans and other ethnic groups: implications for cancer surveillance and risk models. Cancer Epidemiol Biomark Prev.

